# Microbial redox processes in deep subsurface environments and the potential application of (per)chlorate in oil reservoirs

**DOI:** 10.3389/fmicb.2014.00428

**Published:** 2014-09-01

**Authors:** Martin G. Liebensteiner, Nicolas Tsesmetzis, Alfons J. M. Stams, Bartholomeus P. Lomans

**Affiliations:** ^1^Laboratory of Microbiology, Wageningen UniversityWageningen, Netherlands; ^2^Shell International Exploration and Production Inc.Houston, TX, USA; ^3^Center of Biological Engineering, University of MinhoBraga, Portugal; ^4^Shell Global Solutions International B.V.Rijswijk, Netherlands

**Keywords:** oil reservoirs, deep subsurface, anaerobic redox processes, (per)chlorate reduction, nitrate, MEOR, reservoir souring

## Abstract

The ability of microorganisms to thrive under oxygen-free conditions in subsurface environments relies on the enzymatic reduction of oxidized elements, such as sulfate, ferric iron, or CO_2_, coupled to the oxidation of inorganic or organic compounds. A broad phylogenetic and functional diversity of microorganisms from subsurface environments has been described using isolation-based and advanced molecular ecological techniques. The physiological groups reviewed here comprise iron-, manganese-, and nitrate-reducing microorganisms. In the context of recent findings also the potential of chlorate and perchlorate [jointly termed (per)chlorate] reduction in oil reservoirs will be discussed. Special attention is given to elevated temperatures that are predominant in the deep subsurface. Microbial reduction of (per)chlorate is a thermodynamically favorable redox process, also at high temperature. However, knowledge about (per)chlorate reduction at elevated temperatures is still scarce and restricted to members of the Firmicutes and the archaeon *Archaeoglobus fulgidus*. By analyzing the diversity and phylogenetic distribution of functional genes in (meta)genome databases and combining this knowledge with extrapolations to earlier-made physiological observations we speculate on the potential of (per)chlorate reduction in the subsurface and more precisely oil fields. In addition, the application of (per)chlorate for bioremediation, souring control, and microbial enhanced oil recovery are addressed.

## INTRODUCTION

Microorganisms inhabit subsurface environments 100s of meters below Earth’s surface where oxygen is most often lacking. The development of the first modern oil wells in the 19th century opened the “gate to the deep biosphere” and not long after that scientists discovered the first microbes thriving in these environments (Bastin, 1926; Ginzburg-Karagicheva, 1926; Gahl and Anderson, 1928). Particularly the studies of Tausson (1925) and Zobell (1945) gained detailed insight into the microbial oxidation of hydrocarbons by indigenous subsurface microbes. A large number of studies in the following decades tightened the concept of an active and diverse microbial subsurface community. The development of improved anaerobic culturing techniques during the second half of the 20th century resulted in another step forward in the identification of anaerobes and their physiology (Magot et al., 2000; Youssef et al., 2009). These indigenous subsurface microbes were isolated and often deposited in publicly accessible strain collections. A major driver for investigating the microbiology of oil reservoirs has been the biogenic *in situ* formation of hydrogen sulfide from sulfate, causing souring. The detrimental effects associated with the formation of hydrogen sulfide (high toxicity, sulfide stress cracking, corrosion, precipitation of metal sulfides) increase the production and refinery costs of petroleum (Tang et al., 2009) and have created a generally negative image of microorganisms in oil fields from the beginning of modern oil recovery (Bastin et al., 1926). However, particular microorganisms indigenous (or introduced) to the subsurface may have characteristics that are desirable during oil recovery, and it might be beneficial to stimulate these further *in situ*. The most prominent example is the mitigation of souring by nitrate-reducing communities in oil fields (Hubert and Voordouw, 2007; Gieg et al., 2011). Additionally, growing effort is spent on the development of new strategies for microbial enhanced oil recovery (MEOR), or other processes (e.g., conversion of coal to methane) that use the “help of microorganisms” for increasing hydrocarbon recovery.

## SUBSURFACE MICROBIOLOGY

The developments in molecular biology made it possible to obtain a deeper insight into the microorganisms that inhabit oil reservoirs. Numerous studies have been conducted which describe the bacterial and archaeal community structure of produced waters using the 16S rRNA gene marker (clone libraries, DGGE, pyrosequencing; Orphan et al., 2000; Ren et al., 2011; Agrawal et al., 2012). The genomes of an increasing number of subsurface microorganisms have been sequenced and the advances of next generation DNA sequencing technologies have made metagenomic analyses on samples from the subsurface and oil reservoirs possible (Kotlar et al., 2011; An et al., 2013; Lewin et al., 2013). The computational processing and comparison of the steadily growing amount of information in databases will provide a detailed picture of the subsurface microbiota. Nevertheless, cultivation and isolation of microorganisms is indispensable for the characterization of novel enzymes and metabolic pathways and will deepen the interpretability of future sequencing data information.

The authenticity of indigenous microbes isolated from oil fields (and subsurface environments in general) is controversial. Oil fields that have not been treated with secondary recovery methods may be considered pristine, however already the drilling into the formation is a potential source of “microbial contaminations.” Magot (2005) emphasized the additional risk of contamination during sampling and processing of subsurface material. Especially for cultures that differ considerably in growth requirements from the original *in situ* conditions the autochthonous character is often questioned (Magot, 2005). Nevertheless, an unexpected high diversity of aerobic microorganisms was observed in several oil deposits (coal beds and oil sands), where oxygen was assumed to be very limited (An et al., 2013). The study further demonstrated that the presence of the respective aerobes was not attributed to anthropogenically caused contaminations.

Microorganisms thriving in the subsurface are phylogenetically and physiologically diverse. Here, we focus on microbially mediated redox reactions involving terminal electron acceptors that allow energy conservation and growth when coupled to the oxidation of inorganic or organic electron compounds (**Table 1**). The microbial reduction of iron, manganese, nitrate, and (per)chlorate are discussed in this review. Sulfate reducers, methanogens, fermentative, and aerobic microorganisms isolated from oil reservoirs are not covered but these groups of microorganisms were reviewed earlier (Ollivier and Magot, 2005; Youssef et al., 2009).

**Table 1:** Standard reduction potentials of selected redox couples.

Standard reduction potentials (E^0^′) of selected redox couples	
N_2_O/N_2_	+1355 mV
${ClO}_{2}^{-}$/Cl^-^	+1199 mV
2NO/N_2_O	+1175 mV
O_2_/H_2_O	+820 mV
${ClO}_{4}^{-}$/${ClO}_{3}^{-}$	+788 mV
Fe^3+^/Fe^2+^	+772 mV
${ClO}_{3}^{-}$/${ClO}_{2}^{-}$	+709 mV
MnO_2_/Mn^2+^	+380 mV
${NO}_{2}^{-}$/${NH}_{4}^{+}$	+440 mV
${NO}_{3}^{-}$/${NO}_{2}^{-}$	+430 mV
${NO}_{2}^{-}$/NO	+350 mV
${HSO}_{3}^{-}$/HS^-^	-110 mV
CO_2_/CH_4_	-240 mV
${SO}_{4}^{2-}$/${HSO}_{3}^{-}$	-516 mV

*Standard reduction potentials (pH 7; 25°C) were derived from Thauer et al. (1977) and Wolterink (2004).*

### MANGANESE- AND IRON-REDUCING MICROORGANISMS

Microbial ferric iron reduction [Fe(III)] is estimated to have evolved around 3.5 billion years ago and is considered to be one of the oldest respiratory processes on Earth (Richardson, 2000). A relative broad diversity of microorganisms uses ferric iron as electron acceptor. Several iron reducers can also reduce manganese(IV) [Mn(IV)] or other metals (Lovley et al., 2004).

Dissolved or chelated Fe(III) and Mn(IV) are good electron acceptors yielding relatively high amounts of energy (**Table 1**). However, the standard redox potential of the redox couple Fe(III)/Fe(II) (+770 mV) is only relevant at low pH where Fe(III) is soluble. In general, produced water from oil reservoirs is in the range of circumneutral pH. Therefore the concentrations of dissolved Fe(III) is very low and practically unavailable for microbial use (Lovley et al., 2004); same is the case for Mn(IV) and Mn(III). Moreover, the predominant form of Fe(III) and Mn(IV) in subsurface environments is bound in solid minerals (Lovley, 1997). Their use as terminal electron sink for microbial redox reactions is hence associated with less favorable redox potentials and lower accessibility (Thamdrup, 2000). Some microorganisms have physiological adaptations that enable them to utilize insoluble forms of ferric iron as electron acceptors. Several such mechanisms have been described in mesophilic Gram-negative bacteria, particularly for *Geobacter sulfurreducens* and *Shewanella putrefaciens* (Lovley et al., 2004; Shi et al., 2007, 2009; Lovley, 2008). The physiology of Gram-positive and archaeal iron reducers is much less understood (Gavrilov et al., 2012). The first archaeal ferric iron reductase was isolated and characterized from *Archaeoglobus fulgidus* (Vadas et al., 1999; Slobodkin, 2005). Its potential role in energy conservation was discussed, but dissimilatory growth on ferric iron has never been observed (Vadas et al., 1999).

For respiration with ferric iron microbes use organic and inorganic compounds as electron donors (Slobodkin, 2005). A limited number of strains with the ability to reduce soluble and insoluble forms of ferric iron [e.g., *Shewanella putrefaciens* (formerly named *Alteromonas putrefaciens*)] was isolated from oil reservoirs (Semple and Westlake, 1987; Semple et al., 1989). Microbial iron reduction also occurs at high temperature and is wide-spread over the bacterial and the archaeal domain of life (Slobodkin, 2005).

The first thermophilic Fe(III)-/Mn(IV)-reducing microorganism isolated from an oil field was *Deferribacter thermophilus* (Greene et al., 1997; **Table 2**). Slobodkin et al. (1999) isolated numerous other microbial strains from an oil reservoir. These belonged to the genera *Thermoanaerobacter*, *Thermotoga*, and *Thermococcus* and were able to reduce ferric iron. With this finding they concluded that the reduction of ferric iron may be a common trait for energy conservation among anaerobic thermophiles in oil reservoirs.

**Table 2:** Microorganisms isolated from oil field environments, that are able to grow by the reduction of nitrate, Fe(III), and/or Mn(IV).

Species	Strain	Growth [^∘^C], (optimum)	Electron acceptors	Electron donor used	Source	Reference
*Arcobacter* sp.	FWKO B	15–40	Nitrate	Sulfide, hydrogen, formate	Produced brine, oil field (Canada)	Gevertz et al. (2000)
*Deferribacter thermophilus*	BMA^T^	50–65 (60)	Nitrate, Mn(IV), Fe(III)	Hydrogen, malate, acetate, citrate, pyruvate, lactate, succinate, valerate	Production water, North sea oil field (UK)	Greene et al. (1997)
*Denitrovibrio acetiphilus*	N2460^T^	4–40 (35–37)	Nitrate	Acetate	Oil field environment/oil refinery	Myhr and Torsvik (2000)
*Garciella nitratireducens*	MET79^T^	25–60 (55)	nitrate	Lactate, butyrate, malate, fumarate and others	Oil field separator, oil field (USA)	Miranda-Tello et al. (2003)
*Geoalkalibacter subterraneus*	Red1^T^	30–50 (40)	Fe(III), Mn(IV), nitrate	Formate, acetate, propionate, lactate, butyrate, isobutyrate, succinate, fumarate, valerate, isovalerate, citrate, salicylate, octanoate, palmitate, glycerol, hydrogen and others	Produced water, oil field (USA)	Greene et al. (2009)
*Geobacillus lituanicus*	N-3^T^	55–70 (55–60)	Nitrate	Yeast	Oil field (Lithuania)	Kuisiene et al. (2004)
*Geobacillus subterraneus*	34^T^	45–65	Nitrate	Acetate	Formation water, oil field (China)	Nazina et al. (2001)
*Marinobacter hydrocarbonoclasticus* (formerly *M. aqueolei*)	VT8	13–50 (30)	Nitrate	Acetate, succinate, citrate	Produced fluid, oil field (Vietnam)	Huu et al. (1999)
*Petrobacter succinatimandens*	4BON^T^	35–60 (55)	Nitrate	Formate, fumarate, pyruvate, succinate, ethanol, yeast extract	Production water, oil field (Australia)	Salinas et al. (2004)
*Shewanella putrefaciens* (formerly*Alteromonas putrefaciens*)			Fe(III)	Hydrogen, formate	Produced water, oil storage tanks (Canada)	Semple and Westlake (1987)
*Sulfurimonas* sp. (formerly *Thiomicrospira* sp.)	CVO	5–35	Nitrate, nitrite, N_2_O	Sulfide, elemental sulfur	Produced brine, oil field (Canada)	Gevertz et al. (2000)
*Thermoanaerobacter acetoethylicus*	SL 26, S128	40–80 (65)	Fe(III)	Peptone, hydrogen	Formation water, oil field (Russia)	Slobodkin et al. (1999)
*Thermoanaero-bacter brockii*	M739	35–85 (65)	Fe(III)	Peptone, hydrogen	Formation water, oil field (Russia)	Slobodkin et al. (1999)
*Thermococcus sibiricus*	MM 739^T^	40–88 (81)	Fe(III)	Peptone, hydrogen	Formation water, oil field (Russia)	Slobodkin et al. (1999)
*Thermotoga subterranea*	SL-1	50–75 (70)	Fe(III)	Peptone, hydrogen	Formation water, oil field (France)	Slobodkin et al. (1999)

Another oil field isolate that derived from a moderately hot oil field, *Geoalkalibacter subterraneus*, grows by the reduction of Fe(III), Mn(IV), nitrate or elemental sulfur, and trimethylamine-*N*-oxide (Greene et al., 2009).

Iron-reducing microorganisms, besides from oil reservoirs, have also been isolated from other hot environments such as marine and terrestrial hydrothermal vents, hot freshwater springs, and geothermally heated soils [reviewed in Lovley et al. (2004) and Slobodkin (2005)]. Microbial iron reduction has been reported to occur up to 121°C and at salinities 10-times higher than that of sea water (Kashefi and Lovley, 2003; Nevin et al., 2003). Such environmental conditions are also common to hot oil reservoirs.

In turn, the oxidation of ferrous iron in anaerobic environments is an important microbially mediated process, probably innate to nitrate reducers in general (Weber et al., 2006; Carlson et al., 2013).

At circumneutral pH, Fe(II), and Mn(II) are several magnitudes more soluble than their oxidized counterparts [Fe(III), Mn(III), Mn(IV)] (Thamdrup, 2000). To which extent the injection of nitrate in oil fields may result in the oxidation of *in situ* deposited metal(hydr-)oxides is unknown.

A novel MEOR strategy was proposed dosing dissolved ferrous iron together with nitrate (Zhu et al., 2013). The microbial *in situ* formation of solid forms of ferric iron by the action of nitrate-reducing microorganisms could eventually result in improved sweep efficiencies.

### NITRATE-REDUCING MICROORGANISMS

The injection of nitrate during water flooding is applied for souring mitigation purposes, diminishing the biogenic *in situ* formation of hydrogen sulfide by sulfate-reducing prokaryotes (SRP).

In oil fields, nitrate is reduced by microorganisms to di-nitrogen gas (denitrification) or ammonia, using inorganic and organic electron donors (Hubert and Voordouw, 2007). The first step in denitrification and dissimilatory nitrate reduction to ammonia is the reduction of nitrate to nitrite. This conversion is catalyzed by nitrate reductases of the respiratory Nar-type (with the catalytic subunit located in the cytoplasm for bacteria and periplasm for archaea, respectively) and the Nap-type reductases (catalytic subunit in the periplasm; Magalon et al., 2011). Both types of nitrate reductases are found in microorganisms thriving in oil fields (Feng et al., 2007, 2011).

Strain CVO and strain FWKO B, related to the genus *Sulfurimonas* (formerly *Thiomicrospir*a) and *Arcobacter*, respectively, are chemolithoautotrophic nitrate-reducing mesophiles both isolated from produced fluids (Gevertz et al., 2000). Strain FWKO B couples the oxidation of sulfide, hydrogen, or formate and strain CVO the oxidation of sulfide and elemental sulfur to the reduction of nitrate. Whether these microorganisms can couple the reduction of nitrate also to the oxidation of ferrous iron is not known.

One of the heterotrophic nitrate reducers isolated from oil reservoirs is *Deferribacter thermophilus* (Greene et al., 1997). This thermophile in addition to nitrate can also reduce Fe(III) and Mn(IV; see Manganese- and Iron-Reducing Microorganisms). *Geobacillus* is a prominent genus associated with nitrate reduction at elevated temperature in oil reservoirs (Kato et al., 2001; Nazina et al., 2001; Wang et al., 2006). *Geobacillus* species can utilize a broad range of carbon sources. Some isolates are also able to degrade (long)-chain alkanes in the presence of oxygen. (Kato et al., 2001, 2009).

The thermophilic nitrate-reducing oil field isolate *Denitrovibrio acetiphilus* couples the reduction of nitrate to acetate oxidation (Myhr and Torsvik, 2000). *Marinobacter hydrocarbonoclasticus* (synonym *Marinobacter aqueolei*), isolated from an oil reservoir, is a mesophilic bacterium that can grow by the reduction of nitrate and degrades oil compounds under aerobic conditions (Huu et al., 1999).

Numerous thermophilic nitrate reducers have been isolated from other hot environments, like *Thermovenabulum ferriorganovorum* from a hydrothermal vent (Zavarzina et al., 2002), *Garciella nitratireducens* from an oil field separator (Miranda-Tello et al., 2003) or *Caldinitratiruptor microaerophilus* from a hot spring (Fardeau et al., 2010).

*Ferroglobus placidus* was isolated from the vicinity of a hydrothermal vent and is one of the few hyperthermophilic nitrate reducers. It is also able to reduce thiosulfate and Fe(III). This archaeon can also couple the oxidation of aromatic compounds to Fe(III) reduction (Hafenbradl et al., 1996; Tor and Lovley, 2001).

However, to our knowledge no hyperthermophilic nitrate-reducing microorganism has ever been isolated from oil reservoirs up until now. Despite an extensive search on metagenomic resources for hyperthermophilic nitrate reducers, microorganisms like *F. placidus* and *Pyrobaculum aerophilum* are very rarely found to be associated with hydrocarbon resources. Examples include the high temperature (102°C) water samples coming from Bass Strait oil reservoirs where the aforementioned nitrate reducers were detected to be present at very low abundance (0.02 and 0.01%, respectively; MG-RAST ID 4550335.3). Assuming that these microorganisms are indeed present, the lack of nitrate in the water samples as it was reported in the chemical analysis might explain their minor role in this system. Ideally, microbes detected at low abundance should be reported with caution as metagenomics algorithms can sometimes struggle to distinguish between rare microbes and false positives. At the same time, as metagenomic datasets are usually incomplete, failure to detect certain taxa or genes should not be interpreted that they are absent from these particular environments.

## (PER)CHLORATE REDUCERS IN THE DEEP BIOSPHERE?

(Per)chlorate reduction is a well-studied dissimilatory reductive pathway performed under anaerobic conditions (Coates and Achenbach, 2004). The complete reduction of perchlorate (${ClO}_{4}^{-}$) involves the action of a perchlorate reductase (Pcr), that reduces perchlorate to chlorate (${ClO}_{3}^{-}$) and further to chorite (${ClO}_{2}^{-}$) followed by the disproportionation of chlorite to oxygen and chloride by a chlorite dismutase (Cld; Rikken et al., 1996). Microorganisms that carry enzymes which only reduce chlorate but that are incapable of reducing perchlorate are called chlorate reducers (Danielsson Thorell et al., 2003). The respective enzyme, chlorate reductase (Clr) is an enzyme that differs from Pcr in genetic, structural, and evolutionary aspects (Kengen et al., 1999; Bender et al., 2005; Clark et al., 2013).

Most (per)chlorate-reducing microbes favor neutrophilic conditions (Coates et al., 1999) and low salt concentrations. However, some microorganisms have been reported to cope with high salinities during (per)chlorate reduction. *Dechloromarinus chlorophilus* and *Arcobacter* sp. strain CAB can grow at salinities of up to 5 and 3%, respectively (Coates and Achenbach, 2004; Carlström et al., 2013). Actively perchlorate-reducing enrichment cultures were reported at salinities of up to 11% (Logan et al., 2001). Recently, *Marinobacter vinifirmus* and members of the Halobacteriaceae have shown to reduce (per)chlorate at salinities beyond 10% sodium chloride (Xiao and Roberts, 2013; Oren et al., 2014).

The vast majority of (per)chlorate-reducing bacteria are mesophilic facultative anaerobes affiliated to the phylum of Proteobacteria, predominantly belonging to the class of β-Proteobacteria (Coates et al., 1999). Acetate is a common substrate for (per)chlorate reducers, but other organic electron donors were also reported to facilitate (per)chlorate reduction, such as alcohols (Balk et al., 2008, 2010), organic acids (Rikken et al., 1996; Bruce et al., 1999), aromatic hydrocarbons (Coates et al., 2001; Weelink et al., 2008), and aliphatic hydrocarbons (Mehboob et al., 2009). Inorganic electron donors like hydrogen, ferrous iron, zero-valent iron (Son et al., 2006) or thiosulfate, and elemental sulfur (Ju et al., 2008) are also used by (per)chlorate-reducing microbes. For a broad range of mesophilic (per)chlorate-reducing bacteria it was demonstrated that the oxidation of sulfide could be coupled to chlorate and/or perchlorate reduction, resulting in the accumulation of elemental sulfur but not promoting growth (Gregoire et al., 2014). The authors also reported the oxidation of sulfide to sulfate by *Dechloromarinus anomalous* strain NSS during the reduction of chlorate.

### “CLASSICAL (PER)CHLORATE REDUCTION”

Up until now no (per)chlorate-reducing microbes have been isolated from oil reservoirs. Here, we discuss a computational analysis to aid to get insight into the occurrence of (per)chlorate reducers in oil fields.

A crucial necessity for classical (per)chlorate-reducing microorganisms is the presence of a chlorite-disproportionating enzyme, named Cld, that avoids accumulation of the toxic intermediate chlorite. Hundreds of proteins that resemble functional Cld are encoded in phylogenetically diverse groups of prokaryotes (Maixner et al., 2008; Mlynek et al., 2011). The actual number of proteins that are able to catalyze the disproportionation of chlorite to chloride is not known.

Up until now all publicly available genomes of *Geobacillus* species carry a gene encoding a Cld-like protein (pfam06778). Also the genomes of two oil field isolates, *Bacillus cereus* Q1 and *Geobacillus thermodenitrificans* NG80-2 harbor the gene of this Cld-like protein, which is highly conserved within the genus *Geobacillus* (>70% identity over ca. 250 amino acids length). However, its similarity with functionally efficient Clds is low (maximum 24% over full length). The function of this particular protein is unknown, neither has any *Geobacillus* sp. ever been reported to grow by (per)chlorate reduction.

Another subgroup of a Cld-family protein is found in the Halobacteriaceae (>50% homology among different species) and only very remotely related to functional Clds (maximum 23% amino acid sequence identity). Even though this protein group has not been further characterized, several members of the Halobacteriaceae (e.g., *Haloferax mediterranei* and *Haloarcula marismortui*) are able to grow by the reduction of perchlorate and chlorate (Oren et al., 2014). Microorganisms belonging to the genus *Haloferax* and *Halorubrum* (both Halobacteriaceae) were also isolated earlier from oil fields (Zvyagintseva et al., 1995, 1998), however, their ability for (per)chlorate reduction has never been tested. The halobacterial group of Cld-like proteins lacks key residues (Ile88, Trp97, Leu122, Arg127, Glu167 – position refers to *Nitrobacter winogradskyi*) that were identified for functionally active Clds (Mlynek et al., 2011). Based on the same key residues 119 sequences (harbored in 112 microorganisms) were identified from the IMG and related ones from the NCBI database as potentially functional Cld (ranging from lengths of 123–288 amino acids). These sequences belong to a phylogenetically diverse group of mesophiles. Cld-like proteins are assigned according to the earlier proposed lineage I and II (Mlynek et al., 2011; Clark et al., 2013).

The Cld-like protein of *Sedimenticola selenatireducens* for instance has a sequence identity of 65% (with 92% coverage) with the Cld of *Arcobacter* sp. CAB, a known marine (per)chlorate reducer (Carlström et al., 2013). *S. selenatireducens* is an anaerobic selenate-respiring microbe isolated from estuary sediments (Narasingarao and Haggblom, 2006). Although this microorganism has also a Pcr encoded in its genome (gb: ATZE00000000.1) it is not able to grow by the reduction of perchlorate (Narasingarao and Haggblom, 2006).

*Marinobacter manganoxydans*, a halophilic microorganism isolated from a deep-sea hydrothermal vent harbors another Cld-like protein with respective key residues (Mlynek et al., 2011). *M. manganoxydans* is the only genome-sequenced *Marinobacter* species (in total 11; via IMG database) that has a Cld-like protein encoded (gb: EHJ03506.1). *Marinobacter* spp. are ubiquitously found at different depths of the ocean and have been described from oil fields as well (Huu et al., 1999; Singer et al., 2011). Members of the genus *Marinobacter* are able to grow with hydrocarbons as sole carbon and energy source (Duran, 2010). Under anoxic conditions these microorganisms can grow by the reduction of nitrate, using the membrane-bound Nar-type reductase. Studies on the Nar-type reductase of *M. hydrocarbonoclasticus* strain 617 have demonstrated the enzyme’s ability to catalyze chlorate reduction as well (Marangon et al., 2012); a trait known for Nar-type reductases in general (Moreno-Vivian et al., 1999). Another member of the same genus *M. vinifirmus*, was reported to grow by the reduction of nitrate and perchlorate recently (Xiao and Roberts, 2013). The genome of *M. vinifirmus* has not yet been sequenced, however, a Cld similar to the one in *M. manganoxydans* is possibly involved in reducing perchlorate.

Two putative Cld sequences, one deriving from a produced water sample (MHGC; gb: KJ647299; An et al., 2013) and another one from a Canadian oil sand core (gb: KJ647307) were retrieved from metagenomic databases. These partial sequences show resemblance (identity >50%) with the functional Cld of *N. winogradskyi* (ref: YP_319047.1), carrying the key residues of functional lineage II Cld (Mlynek et al., 2011). Sequence KJ647299 (186 amino acids length) is identical to proteins encoded in *Ralstonia pickettii* (Rpic_1480), *Cupriavidus metallidurans* CH34 (Rmet_6340), and *Alicycliphilus denitrificans* BC (Alide_4606); and almost identical to another protein of *A. denitrificans* BC (Alide_4635; 99% identity, 91% query coverage). *A. denitrificans* strain BC is a known chlorate-reducing bacterium. However, another protein in this microorganism was proposed as functional Cld (Alide_4615; Oosterkamp et al., 2013). The proteins Alide_4606 and Alide_4635 on the other hand are both part of a transposon flanking functional enzymes responsible for chlorate reduction located on a plasmid (Clark et al., 2013). Just like the functional Cld of *A. denitrificans* BC the above mentioned proteins are encoded next to Cupin 2 domain genes (Alide_4607 and Alide_4634), which might suggest a functional connection between Cld and Cupin 2 genes (Clark et al., 2013). Alide_4606 and Alide_4635 have high resemblance with the functional Cld of *N. winogradskyi*.

Sequence KJ647307 (with a length of 131 amino acids) is identical to a Cld-like protein encoded in *Pseudomonas stutzeri* A1501 (PST_3351) and very closely related to a hypothetical protein in *P. chloritidismutans* (ref. seq.: WP_023445505.1; 99% identity, 94% query coverage; Wolterink et al., 2002). Several strains of the genus *Pseudomonas* are able to reduce chlorate (Xu et al., 2004; Clark et al., 2013), probably indicating that this trait is more often found in the respective taxon. However, similar to KJ647299 and *A. denitrificans* the resemblance of sequence KJ647307 is not related to the functional Cld of *P. chloritidismutans* (ref: WP_023445619.1).

Due to the ubiquitous distribution of some above discussed microorganisms (e.g., *Marinobacter* spp.) it is likely that they are regularly introduced in off-shore oil reservoirs during the secondary recovery stage of oil recovery. Even in high temperature oil reservoirs some of these mesophilic prokaryotes may survive in the well-bore region where temperatures are lowered by the injected water. Metagenomic analysis on produced fluid samples from oil reservoirs, however, seems to indicate that some of the above mentioned microorganisms (*Pseudomonas*, *Marinobacter*, *Arcobacter*, *Geobacillus*, etc.) might also be indigenous to oil reservoirs.

Even though thermodynamic calculations do not exclude (per)chlorate reduction under elevated temperatures (Amend and Shock, 2001), (hyper)thermophilic (per)chlorate reduction has not been described until recently. The isolation of a thermophilic (per)chlorate-reducing member of the phylum Firmicutes (Balk et al., 2008), *Moorella perchloratireducens* and the recently discovered (per)chlorate-reducing capability of the hyperthermophilic archaeon *Archaeoglobus fulgidus* extended the range of this trait to hot temperatures (Liebensteiner et al., 2013). These findings broadened the diversity of (per)chlorate-reducing prokaryotes considerably. Next to the phylogenetic diversity, also the ecological background of (per)chlorate reducers seems to be wider than previously expected. Whereas *M. perchloratireducens* was isolated from an underground gas storage, the type strain of *A. fulgidus* (strain VC-16) originates from a submarine hot spring (Stetter et al., 1987). *A. fulgidus* strains are, however, also frequently found in subsurface environments like oil reservoirs and deep aquifers, and they are considered to be main contributors to souring in high temperature oil reservoirs (Kaster et al., 2009; Yamane et al., 2011).

### (PER)CHLORATE REDUCTION SENSU LATO IN THE SUBSURFACE

**FIGURE 1:**
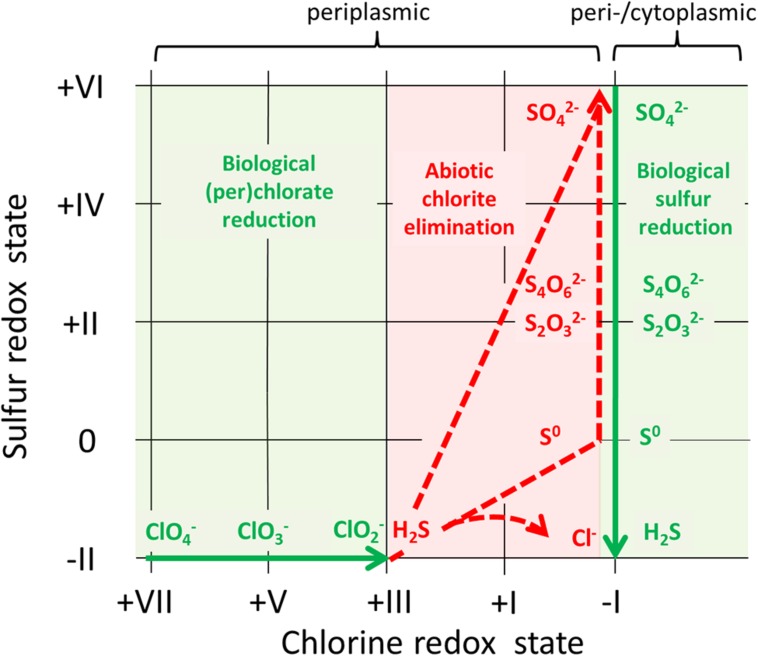
**Complete (per)chlorate reduction by *Archaeoglobus fulgidus* VC-16 involving a biotic–abiotic reaction loop depending on sulfur.** Biological processes are illustrated with green arrows indicating the direction of a respective reaction, whereas dashed red arrows stand for abiotic reactions. Chlorine and sulfur compounds relevant for the complete reduction of perchlorate are shown.

*Archaeoglobus fulgidus* appears to grow by (per)chlorate reduction without the involvement of a Cld. In the absence of a functional Cld an alternative mechanism may enable microorganisms to completely reduce (per)chlorate to chloride anions. In *A. fulgidus* the lack of a chlorite-disproportionating enzyme seems to be overcome by the abiotic scavenging of chlorite formed in the periplasm with naturally occurring or microbially generated sulfide (Liebensteiner et al., 2013). There is indirect evidence that this chemical reaction forms sulfur fractions of higher oxidation states and enables the continuous biological reduction of (per)chlorate. In turn oxidized sulfur compounds are partially reduced back and regenerate reducing power for an ongoing (per)chlorate reduction (**Figure 1**; Liebensteiner et al., 2013).

Similar mechanisms for the biological reduction of (per)chlorate coupled to growth may also occur in reduced subsurface environments (e.g., oil reservoirs) and other microorganisms (besides *A. fulgidus*). Such a “(per)chlorate reduction *sensu lato*” involves an enzyme reducing (per)chlorate, followed by an abiotic chlorite detoxification step.

**FIGURE 2:**
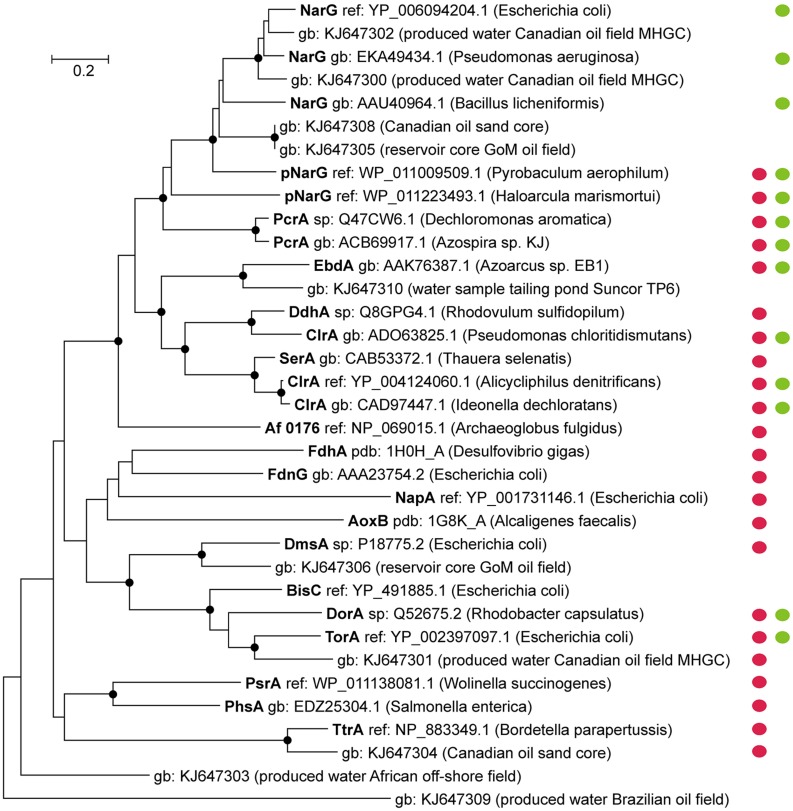
**Diversity of catalytic subunits of selected DMSO Mo-enzymes.** Protein sequences of characterized and functionally active enzymes and partial sequences retrieved from metagenomic datasets of oil reservoir environments are displayed; red circles mark the periplasmic location of the catalytic subunit, whereas green circles indicate activity with chlorate (next to the canonical enzyme function). For most of the DMSO enzymes no data are available regarding their activity towards perchlorate. Accession numbers and the respective microorganism/environment where the sequence derived from are indicated. The phylogenetic tree was constructed using the Neighbor-Joining method including bootstrap values (for 500 replicates). Evolutionary distances of the tree were computed using the Poisson correction method; bootstrap values above 70% are indicated by nodes at the respective branches. The scale bar indicates amino acid substitutions per site.

Several characterized molybdenum enzymes of the DMSO reductase family for instance (**Figure 2**) have shown to be rather unspecific in their substrate range. For some enzymes of this group the reduction of chlorate was demonstrated (besides the canonical function) in biochemical tests (Yamamoto et al., 1986; McEwan et al., 1987, 1991); especially Nar-type reductases seem to reduce chlorate at high rates (Moreno-Vivian et al., 1999; Afshar et al., 2001). The activity for enzymes of the DMSO family towards perchlorate has often not been assessed. An exception is the Nar-type enzyme of *Marinobacter hydrocarbonoclasticus* strain 617, which has a very low activity with perchlorate (Marangon et al., 2012).

For the reduction of (per)chlorate *sensu lato* involving chemical chlorite scavenging the periplasmic localization of the functional enzymes will be of crucial importance to prevent the accumulation of toxic chlorite levels in the cell. Under these conditions chlorite would be better accessible for potential scavengers and thus probably enable continuous (per)chlorate reduction (in the absence of a functional Cld).

Additional sequences, associated to DMSO enzymes, were retrieved from metagenomic projects of oil reservoirs (lengths between 419 and 1199 amino acids) and are plotted in **Figure 2**. Some of these “oil reservoir sequences” are related to known enzymes with chlorate-reducing activity [(p)NarG, TorA] (Yamamoto et al., 1986; Moreno-Vivian et al., 1999) and may form enzymes that are able to reduce chlorate to chlorite. Most of the respective sequences are incomplete, however some carry motifs indicative for a periplasmic location of the protein, using PRED-TAT (Bagos et al., 2010). The presence of (periplasmic) chlorate-reducing enzymes and the reduced character of oil reservoirs (containing sulfide) may enable particular microorganisms to completely reduce chlorate (and perhaps also perchlorate) in the subsurface, similarly to *A. fulgidus*.

## APPLICATION OF (PER)CHLORATE IN THE OIL BUSINESS

Similar to nitrate reduction, the reduction of (per)chlorate involves energetically more-favorable redox couples compared to, e.g., sulfate reduction or methanogenesis (**Table 1**).

The two-step reduction of perchlorate to chlorite via chlorate and its subsequent disproportionation liberates molecular oxygen (van Ginkel et al., 1996). Microbial (per)chlorate reduction is therefore a metabolism potentially forming molecular oxygen under *de facto* anaerobic non-phototrophic conditions. This light-independent *in situ* oxygen production (in contrast to photosynthesis) is exceptional and offers innovative possibilities for applications in the oil recovery business. Unlike oxygen, which is soluble in the mM-range, (per)chlorate is soluble in the M-range.

In a previous study, it was demonstrated that oxygen generated by Cld could even be utilized by other microbes living in a consortium with a (per)chlorate-reducing bacterium (Coates et al., 1998). Given the fact that aerobic processes are energetically more favorable compared to anaerobic ones, the *in situ* formation of oxygen under anaerobic conditions could have promoting effects on both growth yields and rates and thereby allow bioconversion of compounds that are barely degradable under strictly anaerobic conditions.

### BIOREMEDIATION

Man-made perchlorate pollution of soils and drinking water resources is a threat to human health and therefore caused a considerable rise in attention over the last decades. In the 1970s ideas came up to use (per)chlorate-reducing microbes for the purification of (per)chlorate-containing industrial waste waters as well as for the remediation of (per)chlorate-polluted soils (Korenkov et al., 1976). The advances of *in situ* bioremediation of perchlorate-polluted soils have been extensively discussed in the books of Gu and Coates (2006) and Stroo and Ward (2009). A comprehensive review on *ex situ* treatment of perchlorate-containing streams is provided by Sutton (2006).

Besides the bioremediation of toxic perchlorate contamination, the potentially remediating effect of (per)chlorate reduction on the co-degradation of recalcitrant organic pollutants (e.g., hydrocarbons) in anaerobic soil layers was proposed (Coates et al., 1998). The feasibility and extend of *in situ* biodegradation of hydrocarbon-polluted sites often relies on the supply of oxygen. This can be overcome by the injection of compressed air or pure oxygen into deeper anaerobic soil layers but is associated with high costs and a limited penetration of the soil body. (Per)chlorate-reducing bacteria could potentially form oxygen under anaerobic conditions when the intermediate chlorite is disproportionated during the reduction of perchlorate and chlorate. Even though oxygen accumulation has never been observed in cultures growing on (per)chlorate, experiments with washed cell suspensions form and release molecular oxygen upon the addition of chlorite (Rikken et al., 1996; Coates et al., 1998).

For the bioremediation of recalcitrant organic compounds the concept assumes that (per)chlorate reduction results in the release of molecular oxygen under anaerobic conditions that might set on the action of oxygenases, involved in the degradation of pollutants (Jindrova et al., 2002; Wentzel et al., 2007) or be utilized as a terminal electron acceptor coupled to the oxidation of the pollutant. Both would thermodynamically be favored over anaerobic degradation and thus enable higher growth rates and faster degradation rates of pollutants (Tan et al., 2006). This has already been demonstrated with studies on bacterial isolates that reduce (per)chlorate coupled to the oxidation of different aromatic and aliphatic hydrocarbons in pure culture (Weelink et al., 2008; Mehboob et al., 2009). The respective microorganisms had comparable growth rates with chlorate and oxygen during hydrocarbon oxidation.

The broad-scale injection of perchlorate or chlorate to contaminated soils, probably combined with bioaugmentation of (per)chlorate-reducing microbes remains an interesting but debatable strategy due to the potential toxic effects of chlorine oxyanions. The *in situ* applicability of chlorite injections at hydrocarbon-contaminated sites, already containing microorganisms growing on (per)chlorate is tempting (Coates et al., 1998) but can also be questioned. Limitations may be associated with the high toxicity of chlorite at low concentrations and the high chemical reactivity of chlorite with reduced soil or iron particles (Henderson et al., 2001).

### RESERVOIR SOURING MITIGATION

**FIGURE 3:**
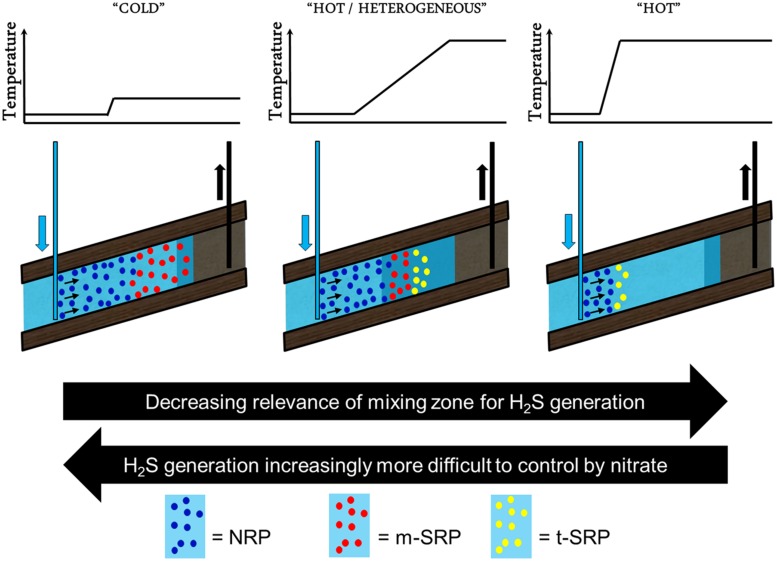
**Schematic representation of water flood with nitrate injection in cold, and hot oil reservoirs with heterogeneous and homogeneous permeability distribution.** The difference in the thermal gradient (graph) and the size of the injection/formation water mixing zone (dark blue) determine zones that are dominated by nitrate- (NRP, blue), mesophilic and (hyper)thermophilic SRP (m-SRP, red and t-SRP, yellow, respectively). Hyperthermophilic nitrate-reducing microorganism like e.g., *Pyrobaculum aerophilum* have not yet been identified in significant numbers in samples derived from oil reservoirs. Nitrate injection does provide protection against souring in the vicinity of the injector well bore in hot reservoirs (right panel), but this protection becomes significantly more challenging in the deeper nitrate-depleted parts of the low-temperature reservoir exposed to sulfate-containing injection or formation water (left panel). Reservoir souring is also strongly dependent on the mixing of nutrients from the formation and injection water and therefore tends to be more extensive in heterogeneous reservoirs (middle panel).

Reservoir souring had long been considered to occur only due to abiotic subsurface processes (Herbert, 1987; Eden et al., 1993; Khatib and Salanitro, 1997). When the role of sulfate-reducing bacteria was acknowledged (Ligthelm et al., 1991; Sunde et al., 1993), this resulted in efforts directed to develop strategies to mitigate microbial reservoir souring. So far, this has resulted in a number of proposed strategies: nitrate injection, sulfate removal and biocide injection. Although probably most effective in the majority of cases, sulfate removal is only scarcely applied for souring mitigation purposes. This is due to the high investment and operational cost associated with sulfate removal units. Application of biocide is used by oil and gas companies to achieve microbial control in their surface production and processing facilities, but it is generally debated whether it is effective to control reservoir souring as its effect does not extend sufficiently deep into the reservoir formations. Nitrate injection is the most widely accepted and used strategy to control microbial reservoir souring, especially effective in hot reservoirs with homogeneous permeability distribution (**Figure 3**; right panel) and to a somewhat lower extend also in ones with a highly heterogeneous permeability distribution (**Figure 3**; middle panel). Nitrate is considered to be effective in controlling reservoir souring by; (1) the competitive exclusion of sulfate-reducing bacteria by more efficient nitrate-reducing bacteria (competing over the same electron donating compounds; volatile fatty acids, BTEX, other hydrocarbons, etc.), (2) inhibition of the dissimilatory sulfite reductase, a key enzyme in the sulfate reduction pathway, by nitrite (an intermediate in reduction of nitrate; Wolfe et al., 1994), and (3) the oxidation of generated sulfide by nitrate-reducing sulfide-oxidizing bacteria (Hubert and Voordouw, 2007). The effectiveness of nitrate injection to control souring is, however, questionable for low-temperature reservoirs. Nitrate might provide protection against souring in the vicinity of the injector well bore, but not in the deeper nitrate-depleted parts of the low-temperature reservoir exposed to injection water containing 30–60 times more sulfate than nitrate (Callbeck et al., 2011, 2013). This would result in the development of zones that are dominated either by nitrate- and sulfate-reducing communities (**Figure 3**; left panel; Voordouw et al., 2009). The success of nitrate injection to control souring in low temperature reservoirs is therefore very much linked to how deep nitrate can be delivered into the reservoir.

Depending on the type and abundance of nitrate- and sulfate-reducing microbes, nitrate might also be less effective in high temperature reservoirs with heterogeneous permeability distribution as these systems might have become depleted for nitrate and tend to have larger high temperature mixing zones where nitrate might not be able to prevent growth of more-temperature tolerant sulfate-reducing microbes (**Figure 3**; middle panel). This seems to be confirmed with the fact that although hyperthermophilic nitrate-reducing microorganism do exist (e.g., *Pyrobaculum aerophilum*), these microorganisms have not yet been identified in significant numbers in samples derived from oil reservoirs (contrarily to hyperthermophilic sulfate reducers).

The limitations of above mentioned strategies are the driver to seek for alternative mitigation strategies. Based on findings that (per)chlorate inhibits sulfate reduction by *A. fulgidus* (Liebensteiner et al., 2013), appears to be toxic to other sulfate reducers (Engelbrektson et al., 2014), and serves as an electron acceptor for sulfide oxidation (Gregoire et al., 2014), (per)chlorate may be an effective alternative to nitrate to mitigate souring with application in both high and low temperature reservoirs. Whereas the biological oxidation of sulfide to elemental sulfur seems to be a characteristic innate to classical (per)chlorate reducers of low temperature optimum, there is a strong indication that the oxidation of sulfide during (per)chlorate reduction at higher temperatures such as that of *A. fulgidus*, is chemically mediated.

In contrast to a mesophilic (per)chlorate-reducing community that doesn’t directly affect the sulfate-reducing community and would have to be established first, chlorate reduction by *A. fulgidus* appeared to have a direct negative impact on the microorganism’s sulfate-reducing capability (Liebensteiner et al., 2013). The increased expression of stress proteins in *A. fulgidus* when exposed to (per)chlorate indicates that the inhibition might be linked to redox stress from the chlorite produced as intermediate.

Given the ability of *A. fulgidus* to reduce (per)chlorate and the fact that chlorate reduction seems to interfere with sulfate reduction, (per)chlorate injection could provide control in high(er) temperature zones where that is not feasible by nitrate. The fact that *Azospira suillum* only oxidized sulfide coupled to (per)chlorate reduction, but not nitrate (which is normally also used together with organic electron donors; Gregoire et al., 2014) may further indicate the different impact of (per)chlorate compared to nitrate during souring mitigation interventions. In other words the alternating use of nitrate and (per)chlorate possibly combined with biocides could make use of complementary effects that avoid scavenging of nitrate/(per)chlorate in the vicinity of the injector well bore and thereby extending the impact of both nitrate as well as (per)chlorate deeper into the reservoir.

### MICROBIAL ENHANCED OIL RECOVERY

**FIGURE 4:**
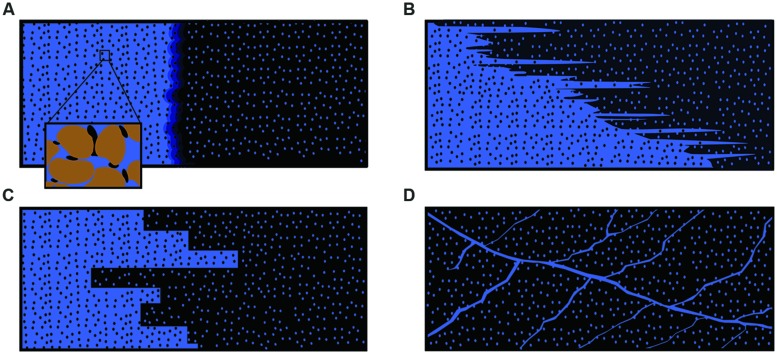
**Schematic representation of oil displacement in a petroleum reservoir.** Ideal matrix piston-wise (stable) displacement leaving low residual oil levels **(A)** with the close-up showing the residual oil (black blobs) attached to sand grain particles (brown), unstable displacement showing fingering of water into the oil phase **(B)**, unstable displacement due to thief zones **(C)** and unstable displacement through fractures (especially for carbonates; **D**). Oil phase is indicated in black, water in blue. Small black blobs indicated residual oil after being flooded, small blue blobs indicate connate water before being flooded.

The efficiency of oil recovery from oil reservoirs is very often limited due to the geological structure of the oil-bearing formation and the oil characteristics. Although a matrix, piston-wise displacement of the target oil is intended (**Figure 4A**), the actual displacement is often highly unstable due to fingering of water in oil (because of viscosity differences; **Figure 4B**) or preferred flow through high permeable zones (**Figure 4C**) or fractures (**Figure 4D**). MEOR has already been proposed at the advent of modern oil production (Beckman, 1926). Although several MEOR trials have been reported and hundreds of patents are filed, the process often lacks reproducibility or remains unproven (Brown, 2010; Bachmann et al., 2014). Moreover, most of the MEOR trials are in fact well stimulation rather than “full-field” MEOR treatments. Many driving mechanisms for MEOR were postulated, of which the *in situ* generation of biosurfactant received lots of attention. Convincing evidence that *in situ* microbes will be able to generate sufficient amounts of effective surfactant in a full-field setting in order to increase the capillary number sufficiently such that residual oil is indeed mobilized is, however, still lacking. A critical analysis of the proposed mechanistic drivers for MEOR revealed that only the plugging of high-permeability zones (aka conformance control), seemed to be most plausible (Gray et al., 2008). In order to be feasible for a field-wide application, an MEOR process based on conformance control would have to rely on the stimulation of indigenous microbes (avoiding requirement of injecting microbes) utilizing part of the hydrocarbon fraction (or *in situ* commonly occurring volatile fatty acids) as electron donor. The reduction of (per)chlorate in the subsurface might liberate highly oxidative chlorine intermediates or even oxygen in a *de facto* anaerobic environment. Reactive chlorine oxyanions (chlorite) and oxygen may either chemically or biologically oxidize (in)organic compounds (e.g., sulfide, ferrous iron, hydrocarbons etc.) in the vicinity of the (per)chlorate reducer. The availability of oxygen is also a crucial pre-requisite for hydrocarbon-utilizing mono- and dioxygenases. These may yield “activated hydrocarbons” that are subsequently more easily degradable by (other) microorganisms. The presence of oxygen would enable facultative prokaryotes to switch from a lower-efficiency anaerobic “lifestyle” to a more efficient microaerophilic metabolism, generating more biomass. We therefore propose that injection of (per)chlorate alone or in combination of nitrate and phosphates (if the latter proves to be limiting), might be able to sufficiently stimulate the indigenous microbial community to achieve conformance control and thereby enhance oil production. Further research is needed to show the effectiveness of (per)chlorate injection for MEOR.

## Conflict of Interest Statement

The authors declare that the research was conducted in the absence of any commercial or financial relationships that could be construed as a potential conflict of interest.
